# A COVID-19 Pandemic Artificial Intelligence–Based System With Deep Learning Forecasting and Automatic Statistical Data Acquisition: Development and Implementation Study

**DOI:** 10.2196/27806

**Published:** 2021-05-20

**Authors:** Cheng-Sheng Yu, Shy-Shin Chang, Tzu-Hao Chang, Jenny L Wu, Yu-Jiun Lin, Hsiung-Fei Chien, Ray-Jade Chen

**Affiliations:** 1 Department of Family Medicine School of Medicine, College of Medicine Taipei Medical University Taipei Taiwan; 2 Department of Family Medicine Taipei Medical University Hospital Taipei Taiwan; 3 Graduate Institute of Biomedical Informatics College of Medical Science and Technology Taipei Medical University Taipei Taiwan; 4 Professional Master Program in Artificial Intelligence in Medicine College of Medicine Taipei Medical University Taipei Taiwan; 5 Clinical Big Data Research Center Taipei Medical University Hospital Taipei Taiwan; 6 Division of Plastic Surgery, Department of Surgery Taipei Medical University Hospital and School of Medicine, College of Medicine Taipei Medical University Taipei Taiwan; 7 Department of Surgery School of Medicine, College of Medicine Taipei Medical University Taipei Taiwan; 8 Division of General Surgery Department of Surgery Taipei Medical University Hospital Taipei Taiwan

**Keywords:** COVID-19, artificial intelligence, time series, deep learning, machine learning, statistical analysis, pandemic, data visualization

## Abstract

**Background:**

More than 79.2 million confirmed COVID-19 cases and 1.7 million deaths were caused by SARS-CoV-2; the disease was named COVID-19 by the World Health Organization. Control of the COVID-19 epidemic has become a crucial issue around the globe, but there are limited studies that investigate the global trend of the COVID-19 pandemic together with each country’s policy measures.

**Objective:**

We aimed to develop an online artificial intelligence (AI) system to analyze the dynamic trend of the COVID-19 pandemic, facilitate forecasting and predictive modeling, and produce a heat map visualization of policy measures in 171 countries.

**Methods:**

The COVID-19 Pandemic AI System (CPAIS) integrated two data sets: the data set from the Oxford COVID-19 Government Response Tracker from the Blavatnik School of Government, which is maintained by the University of Oxford, and the data set from the COVID-19 Data Repository, which was established by the Johns Hopkins University Center for Systems Science and Engineering. This study utilized four statistical and deep learning techniques for forecasting: autoregressive integrated moving average (ARIMA), feedforward neural network (FNN), multilayer perceptron (MLP) neural network, and long short-term memory (LSTM). With regard to 1-year records (ie, whole time series data), records from the last 14 days served as the validation set to evaluate the performance of the forecast, whereas earlier records served as the training set.

**Results:**

A total of 171 countries that featured in both databases were included in the online system. The CPAIS was developed to explore variations, trends, and forecasts related to the COVID-19 pandemic across several counties. For instance, the number of confirmed monthly cases in the United States reached a local peak in July 2020 and another peak of 6,368,591 in December 2020. A dynamic heat map with policy measures depicts changes in COVID-19 measures for each country. A total of 19 measures were embedded within the three sections presented on the website, and only 4 of the 19 measures were continuous measures related to financial support or investment. Deep learning models were used to enable COVID-19 forecasting; the performances of ARIMA, FNN, and the MLP neural network were not stable because their forecast accuracy was only better than LSTM for a few countries. LSTM demonstrated the best forecast accuracy for Canada, as the root mean square error (RMSE), mean absolute error (MAE), and mean absolute percentage error (MAPE) were 2272.551, 1501.248, and 0.2723075, respectively. ARIMA (RMSE=317.53169; MAPE=0.4641688) and FNN (RMSE=181.29894; MAPE=0.2708482) demonstrated better performance for South Korea.

**Conclusions:**

The CPAIS collects and summarizes information about the COVID-19 pandemic and offers data visualization and deep learning–based prediction. It might be a useful reference for predicting a serious outbreak or epidemic. Moreover, the system undergoes daily updates and includes the latest information on vaccination, which may change the dynamics of the pandemic.

## Introduction

In December 2019, the first cases of a new respiratory disease caused by a novel coronavirus were reported in Wuhan, Hubei province, China [1]. The novel coronavirus was subsequently identified and named SARS-CoV-2, and the disease caused by SARS-CoV-2 was named COVID-19 by the World Health Organization (WHO) [2,3]. Since the time the first cases were reported, many confirmed cases have been reported in various other countries. By March 11, 2020, more than 118,000 confirmed cases and 4291 deaths had been reported across 114 countries. The WHO declared the COVID-19 outbreak a pandemic [4], which continues to worsen. As of December 27, 2020, there were more than 79.2 million confirmed cases and 1.7 million deaths [5]. COVID-19 management has emerged as an urgent global issue. Many studies have investigated the factors that contribute to the spread of COVID-19. Demographic, geographic, and economic factors have influenced the spread of the disease. However, social factors, especially governmental response to the pandemic, have significantly influenced disease severity within certain countries [6-11]. Some countries have shown that implementing rigorous public health care management strategies can successfully control infection spread and maintain normal societal functioning [11].

The rapid development of artificial intelligence (AI) in the health care field offers new opportunities to medical researchers. There are many studies that employ AI techniques in disease predictions, such as Yu et al, who have established an online machine learning health assessment system for metabolic syndrome and chronic kidney diseases [12]. Lin et al utilized multicenter data to develop an end-stage liver disease mortality prediction scoring system [13]. Ayyoubzadeh et al analyzed the rate of COVID-19 incidence in Iran using Google Trends data and deep learning methods [14]. Yeung et al combined several online COVID-19 data to train and evaluate five non–time series machine learning models in predicting confirmed infection growth [15]. These studies have shown that AI is suitable for evaluating disease trends and can provide governments with information that can be used to prevent outspread. There are abundant research findings on COVID-19–related AI prediction and the utilization of mobile sensor data with cell broadcast to identify and manage potential contacts [14,16-20].

However, most of these studies have been conducted in a specific region or single country. There is public health consensus that vaccination is an effective prevention strategy. However, with regard to its efficiency and medical expenditure, long-term follow-up investigation is needed to evaluate the clinical effects of vaccines that have not undergone the standard approval process and tests of their mid- and long-term side effects on different groups [21]. Moreover, different studies have focused on different time frames in pandemic trend prediction. They have drawn the same conclusion: there is a high possibility that COVID-19 will remain a common illness or become endemic in the future, and we must learn to coexist with it. Many factors influence how the pandemic will progress (eg, herd immunity), and governmental and individual responses vary widely across nations [22,23]. Successful epidemic prevention and control measures remain the most efficient solution for public health problems. However, there is limited literature on the relationship between governmental responses and the severity of the domestic spread of COVID-19 [24,25].

Therefore, we constructed an online AI system that contains worldwide COVID-19–related data, each country’s governmental responses to the COVID-19 pandemic, and each country’s population data [26]. The COVID-19 Pandemic AI System (CPAIS) can be used to analyze the dynamic trend of the COVID-19 pandemic, facilitate forecasting and predictive modeling, and produce heat map visualization of policy measures in different countries.

## Methods

### Data Acquisition and System

The CPAIS integrated two data sets: the data set from the Oxford COVID-19 Government Response Tracker (OxCGRT) from the Blavatnik School of Government, which is maintained by the University of Oxford, and the data set from the COVID-19 Data Repository, which was established by Johns Hopkins University Center for Systems Science and Engineering (CSSE). The COVID-19 Data Repository also contains each country’s population data, which are obtained from the United Nations World Population Prospects [27-31]. A total of 171 countries that featured in the databases were included in the system.

The CPAIS was placed on a sever and embedded with time series deep learning models to provide forecasting analyses by the statistical program R, version 3.6.3 (The R Foundation). We used the React.js, version 16.14.0, framework; the styling language Sass (Syntactically Awesome Style Sheets), version 4; and the programming language JavaScript ES6 for front-end implementation. As for back-end implementation, we used Java 8; Spring Boot, version 2.0.2 (VMware, Inc); and R as the programming languages, and we used the MySQL (Structured Query Language), version 5.7.21, database as the storage system. In addition, this AI-based system has been programmed to update itself by auto-retrieving information from all data sets each morning at 9 AM (GMT + 8). The auto-retrieval can be summarized in the following three steps: (1) setting the crawler to fetch the data from the source databases, (2) integrating the updated data into our own MySQL database, and (3) conducting statistical analysis using the database-stored procedure.

The COVID-19 Data Repository established by Johns Hopkins University CSSE contains three categories of data concerning COVID-19 incidence—confirmed cases, recovered cases, and number of deaths—with country geolocation retrieved from 192 affected countries since January 21, 2020. For most of the countries, country-level data concerning the numbers of reported cases are available. Province- and city-level data concerning reported cases are available for some countries. To depict the COVID-19 pandemic comprehensively, we archived country-level data. The number of reported cases was updated daily using data retrieved from multiple online sources. The number of cases was retrieved from the WHO and the regional and local health departments of the affected countries, including their centers for disease control and prevention. All data were shared freely through GitHub.

OxCGRT has been collecting and documenting governmental responses to the COVID-19 pandemic based on several parameters since January 1, 2020. The data set includes 183 countries and 20 items (19 indicators and 1 free response) that characterize governmental responses. There are three types of items: (1) ordinal scale for severity or intensity, (2) numeric scale for specific numbers, and (3) text for other information types. These items can be further classified into four groups: (1) containment and closure policies (8 indicators), (2) economic policies (4 indicators), (3) health system policies (7 indicators), and (4) miscellaneous policies (1 free response). Miscellaneous policies were not included in this system because they were assessed using a free-text response format and limited data were available. OxCGRT data were retrieved from publicly available sources and regularly updated on GitHub.

### Statistical Analysis and Deep Learning Techniques

#### Overview

**Figure 1:**
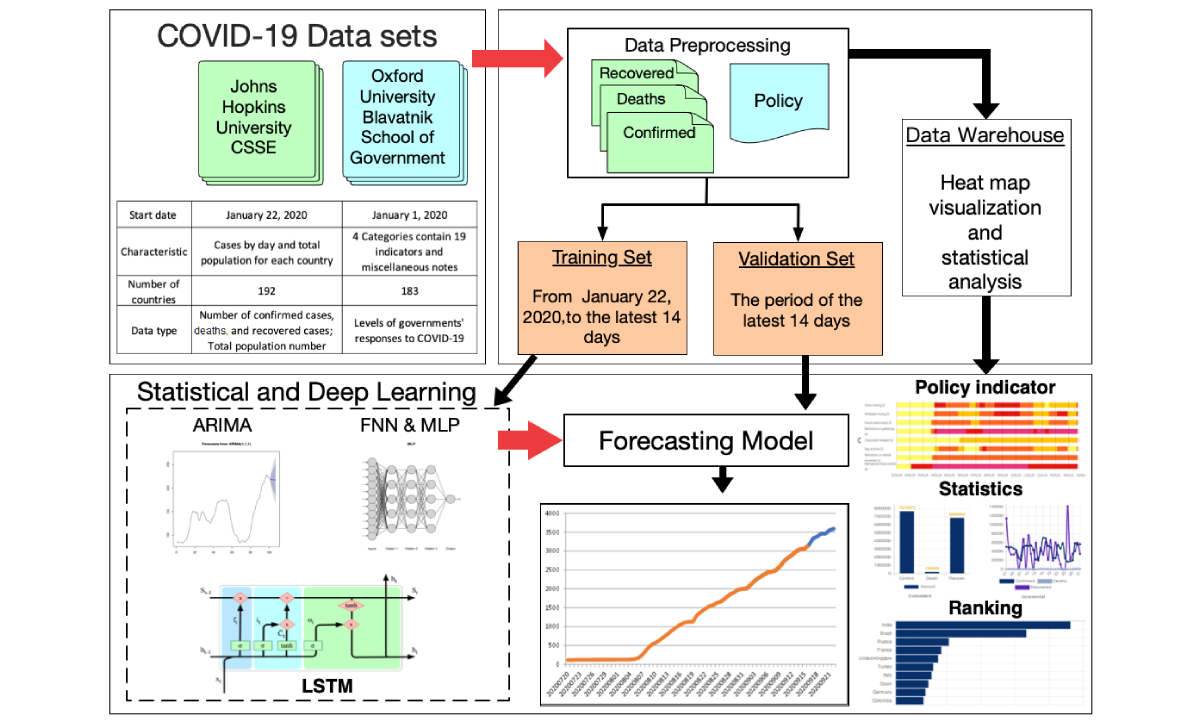
The structure of the COVID-19 Pandemic AI System (CPAIS). ARIMA: autoregressive integrated moving average; CSSE: Center for Systems Science and Engineering; FNN: feedforward neural network; LSTM: long short-term memory; MLP: multilayer perceptron; NN: neural network.

Four time series models were considered for this study. Each model was applied to all the countries in our system to facilitate forecasting. With regard to 1-year records (ie, whole time series data), records from the last 14 days served as the validation set, whereas earlier records served as the training set. Using records from the last 14 days, forecasting performance was evaluated based on the following five indices: mean error (ME), root mean square error (RMSE), mean absolute error (MAE), mean percentage error (MPE), and mean absolute percentage error (MAPE) [32,33]. RMSE, MAE, and MAPE are always positive values, whereas RMSE, MPE, and MAPE are scaled measures. The hyperparameters for each model can be found in Table S1 in Multimedia Appendix 1, and the diagram of the neural networks can be found in Figure 1. R, version 3.6.3 (The R Foundation), was used to conduct statistical analysis and apply deep learning techniques.

#### Autoregressive Integrated Moving Average

An autoregressive integrated moving average (ARIMA) model is a statistical regression analysis that utilizes time series data to either understand the data set better or predict future trends. The purpose of ARIMA is to forecast future trends by examining differences between values in the series rather than by using actual values [34,35]. The three main components of ARIMA are autoregression, integration, and moving average. Autoregression refers to a model with a changing variable that regresses on its lag values. Integration represents the differences between data values and their previous values for stationary time series. Moving average incorporates the dependence between an observation and an error term from a moving average model. An ARIMA model can be comprehended by outlining each component, which serves as a parameter with a standard notation. For ARIMA models, there are three standard notations, wherein integer values serve as substitutes for the parameters to indicate the type of ARIMA model used.

The parameters can be defined as follows:

- p: the number of time lags
- d: the degree of differencing
- q: the size of the moving average window.

In this study, we used the *auto.arima* function for R, which returns the best ARIMA model based on either the Akaike information criterion value or Bayesian information criterion value. The function searches for possible models within the order constraints provided in the *forecast* package for R [36,37].

#### Feedforward Neural Network

A feedforward neural network (FNN) is the simplest type of artificial neural network [38]. The FNN algorithm is biologically inspired. It consists of several simple neuron-like units that are organized in layers. In FNN, information moves in one direction—from the input nodes, through the hidden nodes, and to the output nodes. The mechanism of an FNN is different from that of recurrent neural networks (RNNs) in that connections between the units do not form cycles or loops in FNNs [38,39]. In this study, we used the *nnetar* function for R, which constructs FNNs with a single hidden layer and lagged inputs for the purpose of forecasting univariate time series. Also, in the *forecast* package, the function fits into a single hidden-layer neural network for forecasting, with the *nnet* function included in the *nnet* package for R [40,41].

#### Multilayer Perceptron Neural Network

Like FNNs, multilayer perceptron (MLP) neural networks are common deep learning feedforward networks. An MLP neural network is also a supervised learning algorithm used for classification. The main difference is that between the input and output layer, there can be multiple nonlinear layers, called hidden layers, which are the true computational engine of the MLP neural network. MLP neural networks use a learning technique called back-propagation for training. Their multiple layers and nonlinear activation distinguish MLP neural networks from a linear perceptron [42-44]. In other words, MLP neural networks are designed to solve nonlinearly separable problems. Specifically, the units of MLP neural networks apply a sigmoid function as an activation function. In the back-propagation technique, the difference between the output values and the *ground truth* answer are calculated using predefined error functions. The error is fed back through the network. Using this information, the algorithm can adjust the weights of each connection to significantly reduce the value of the error function. In this study, the *mlp* function fits MLP neural networks for time series forecasting executed using the *nnfor* package [45-47].

#### Long Short-term Memory

Long short-term memory (LSTM) networks are a special type of recurrent deep learning neural network that learns order dependence in sequence prediction problems. LSTM was introduced by Hochreiter and Schmidhuber in 1997, and it is now widely used in a variety of studies and projects [48,49]. A typical RNN makes use of sequential information. These networks are described as *recurrent* because they use their internal state to process the variable length sequences of inputs. It is difficult for a standard RNN to carry forward information from prior time steps to later ones if a sequence is too long, because it may exclude important information from the beginning. Therefore, LSTM has an advantage in that information can be remembered for long periods of time. Unlike traditional FNNs, LSTM has feedback connections, whereby the output from the previous step is supplied as input in the current step [50]. A common LSTM unit includes a cell, an input gate, an output gate, and a forget gate. The cell recalls values over an arbitrary time interval, and the three gates regulate the flow of information in and out of the cell. In this study, we used the *keras* R package to recall TensorFlow for conducting the LSTM analysis [51]. TensorFlow was developed by the Google Brain team and released in 2015. It is a free open-source software library for machine learning techniques, particularly deep neural networks [52].

### Data Visualization of Time Series Data Sets

Heat maps can be generated to depict variations in policy measures for the COVID-19 pandemic across time. Gradient color bars represent changes in measures across different levels and the support received in the form of financial assistance and investments. The time schedule presented along the horizontal axis will be updated daily. Cumulative and monthly records are represented using histograms and line charts, respectively. This system also provides a download option to interested countries and comparable services with dynamic rankings of the total number of confirmed cases and deaths and declining trends for the COVID-19 pandemic. The following simple regression formula is used to examine declining trends with dynamic time intervals:

*y_i_* = *α* + *βx_i_*

where *β* is the slope that represents an increasing or decreasing trend.

## Results

In this study, the CPAIS was developed to explore variations, trends, and forecasts related to the COVID-19 pandemic across several counties. A drop-down list for country selection is available. The framework of the CPAIS—from data acquisition and preprocessing to deep learning model application, forecasting, and data visualization—is presented in Figure 1. It includes a combination of two data sets, construction of databases for deep learning prediction and statistical analysis, four statistical or deep learning models for forecasting, and front-end functions for data visualization.

**Figure 2:**
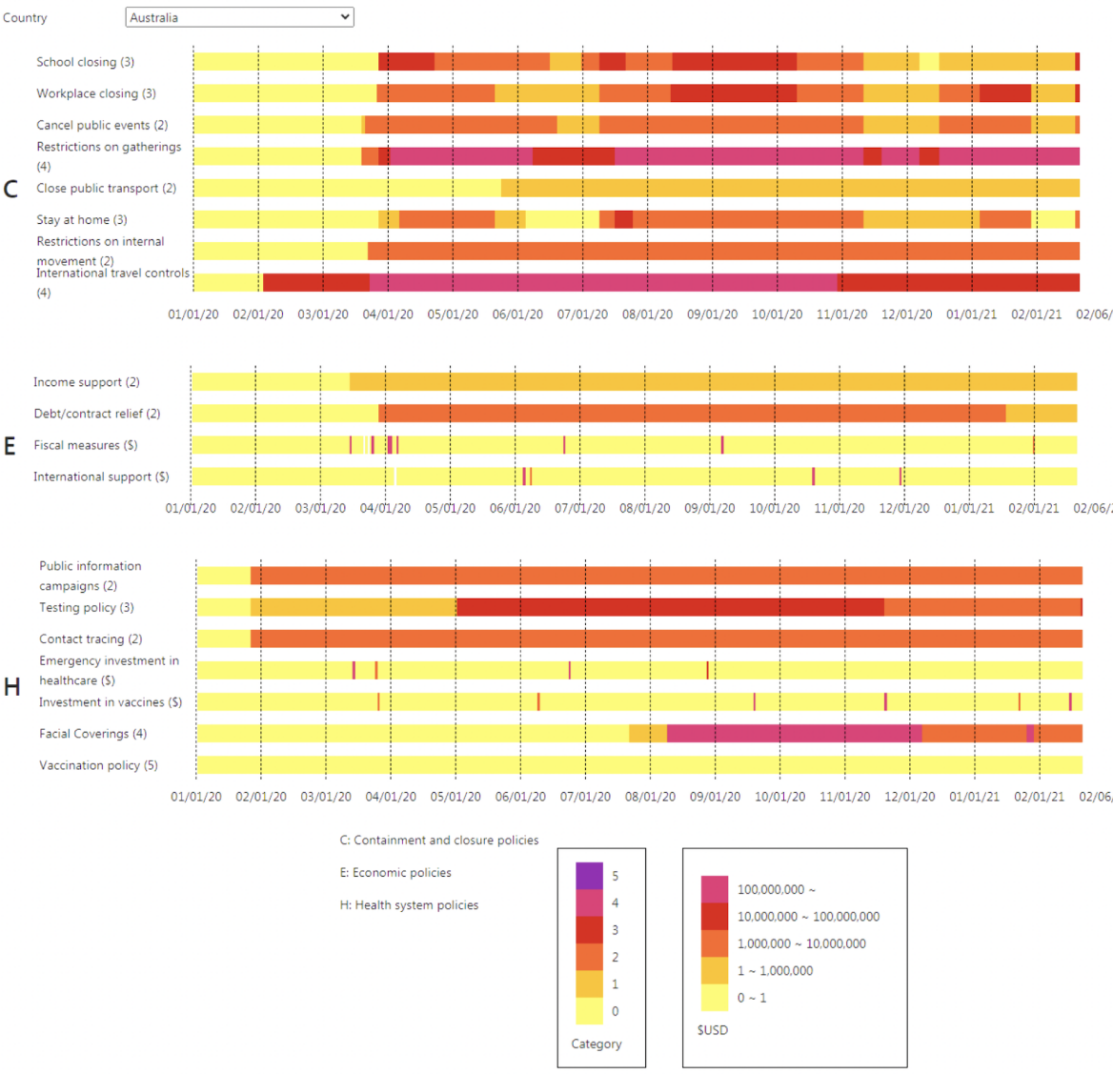
The interface of the dynamic heat map with policy measures on the COVID-19 Pandemic AI System (CPAIS) website.

The numbers of confirmed cases, recovered individuals, and deaths in 15 countries are listed by month in Table 1. The number of confirmed monthly cases in the United States reached a local peak in July 2020 and another peak of 6,368,591 in December 2020. Regarding the United States, the number of recovered cases after December 14, 2020, is not recorded in the COVID-19 Data Repository database. The total population for each of the 15 countries in 2020 is also mentioned in the table. The dynamic heat map with policy measures is shown in Figure 2, which depicts changes in COVID-19 measures for each country, with Australia used as an example. A total of 19 measures were embedded within the three main policy sections (ie, containment and closure policies, economic policies, and health system policies). Economic policies have the least number of measures, and only 4 of the 19 measures are continuous measures related to financial support or investment.

**Figure 3:**
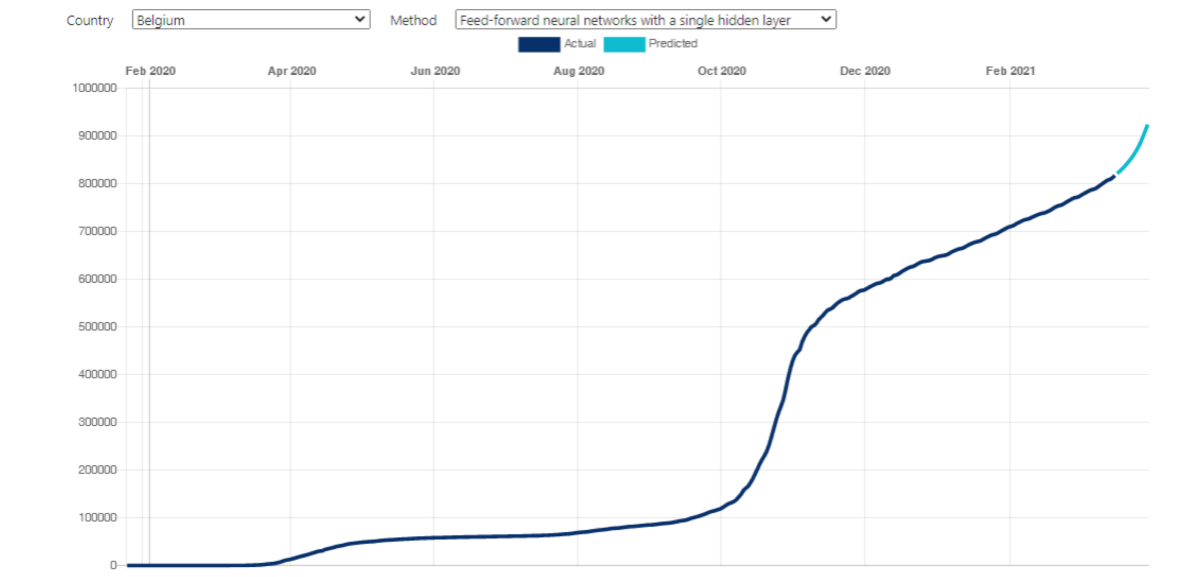
The COVID-19 Pandemic AI System (CPAIS) interface for machine learning prediction models facilitating 14-day COVID-19 forecasting. The plot shows the curve for deep learning modeling of total cumulative confirmed cases.

Deep learning and statistical learning models were used to enable COVID-19 forecasting. The function facilitates 14-day forecasting using four powerful algorithms (Figure 3). ARIMA is the statistical learning model with time series regression; the other models are deep learning neural network algorithms with a single hidden layer, multiple hidden layers, or recurrent techniques. The performance of forecasting for each model for the 15 countries listed in Table 1 is shown in Table 2. A small error value indicates a perfect fit for the data, but the comparison between the different countries was not meaningful because they had different baselines based on their populations. For most of the countries, LSTM demonstrated better forecast accuracy with fewer errors than the other models. The performances of ARIMA, FNN, and the MLP neural network were not stable because their forecast accuracy was only competitive with LSTM for some specific countries. For example, LSTM demonstrated the best forecast accuracy for Canada. The RMSE, MAE, and MAPE were 2272.551, 1501.248, and 0.2723075, respectively. ARIMA (RMSE=317.53169; MAPE=0.4641688) and FNN (RMSE=181.29894; MAPE=0.2708482) demonstrated better performance for South Korea.

**Figure 4:**
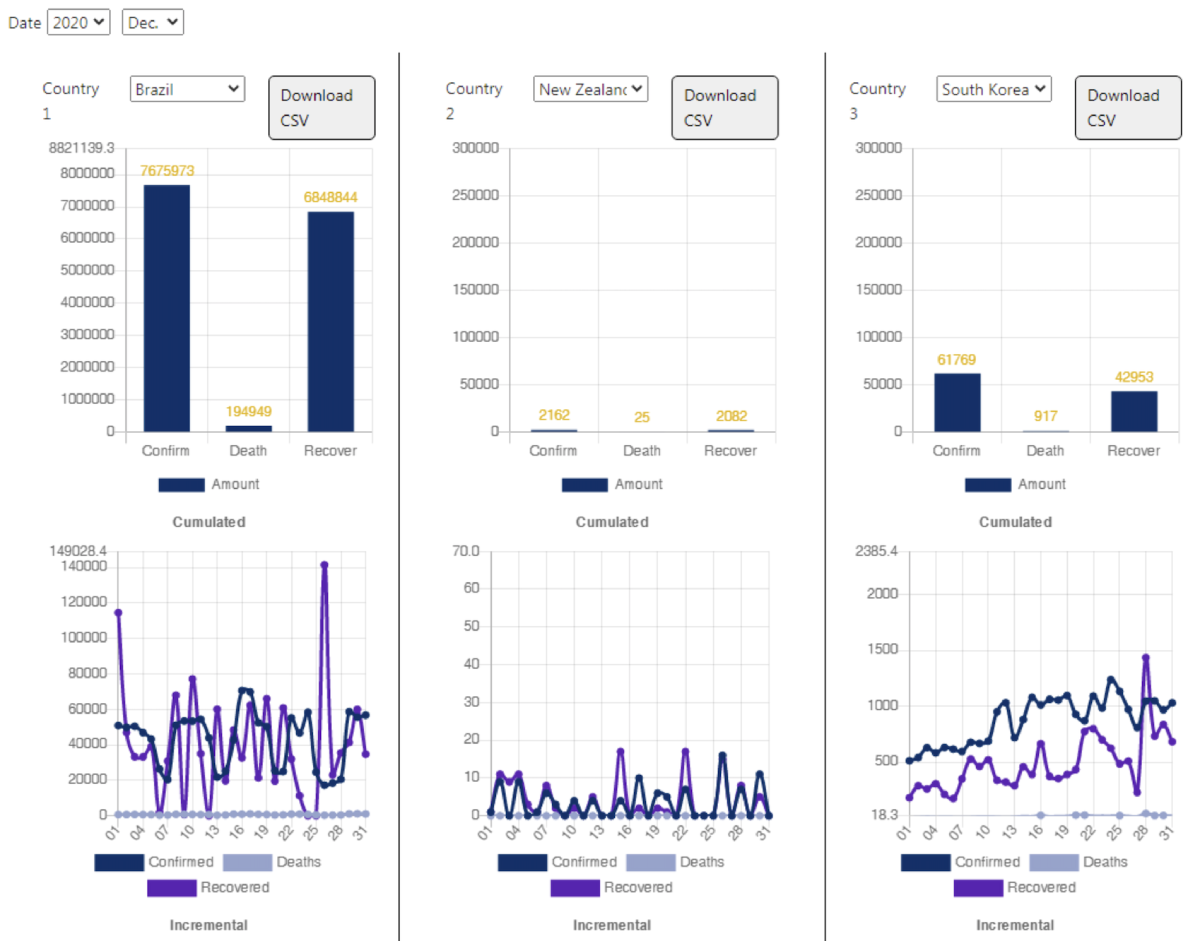
The interface of descriptive statistics for selected countries with customization on the COVID-19 Pandemic AI System (CPAIS) website. CSV: comma-separated values.

Figure 4 presents descriptive statistics for specific countries. On the website, three countries can be simultaneously compared, and the period can be customized. Users can select the countries that are of interest to them and compare the COVID-19–related data. For each respective country, a line chart showing the number of confirmed cases, recoveries, and deaths per month is generated. In addition, a global comparison is also provided on the website.

**Figure 5:**
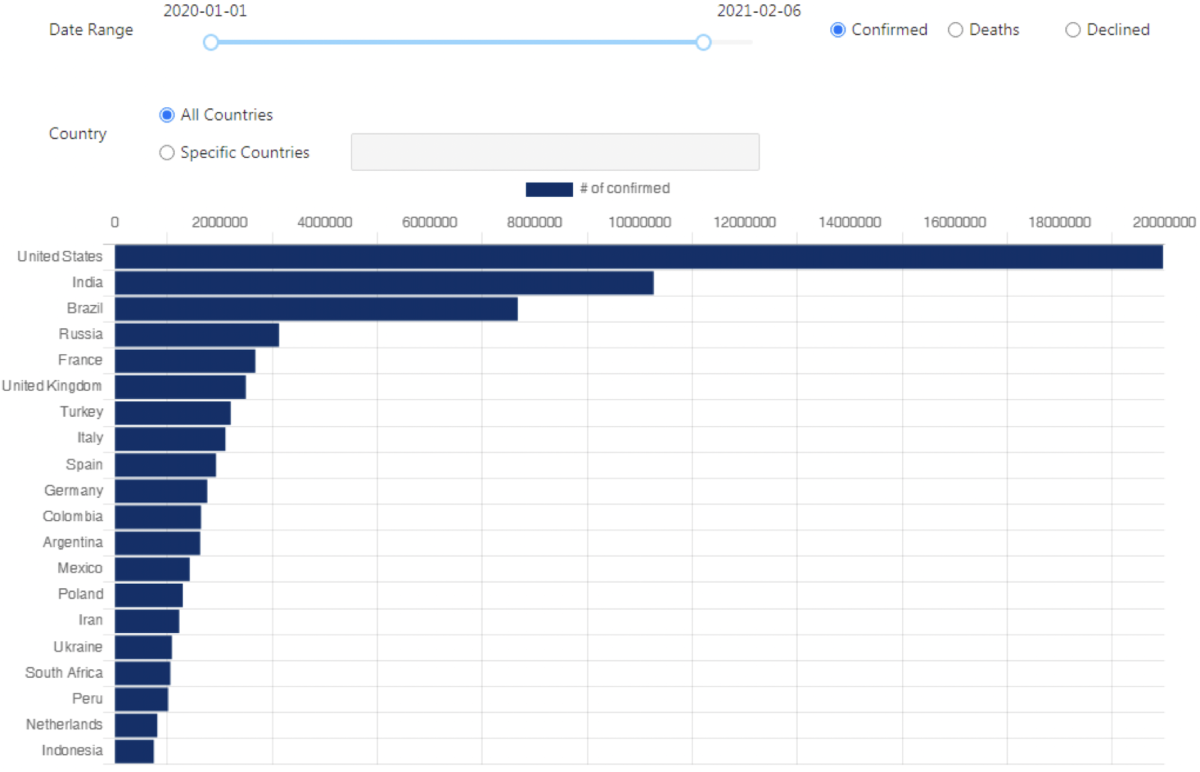
The interface for the ranking of selected countries with customization on the COVID-19 Pandemic AI System (CPAIS) website.

Users can rank 171 countries based on five different parameters: (1) the number of confirmed cases, (2) confirmed cases by percentage of population, (3) the number of confirmed deaths, (4) confirmed deaths by percentage of population, and (5) declining trend. Figure 5 shows an example of how the top 20 countries can be ranked using confirmed cases by percentage of population. With regard to customization, the ranking function is flexible. The selected countries and specific time period can be changed by the user.

**Table 1:** The numbers of confirmed cases, recovered individuals, and deaths in 15 countries by month in 2020.

Country (total population^a^) and cases			Jan		Feb		Mar		Apr		May		June		July		Aug		Sept		Oct		Nov		Dec
**United States (N=329,466,283)**																									
	Confirmed	7		17		192,152		884,047		718,241		834,359		1,922,730		1,464,676		1,201,822		1,914,993		4,466,451		6,368,591	
	Deaths	0		1		5271		60,699		41,703		20,113		26,306		29,591		23,515		23,928		37,038		77,572	
	Recovered	0		7		7017		146,923		290,811		275,873		717,529		746,665		655,863		771,790		1,533,841		1,151,763^b^	
**Canada (N=37,855,702)**																									
	Confirmed	4		16		8507		45,930		38,022		13,618		12,184		12,637		30,189		76,206		144,244		202,852	
	Deaths	0		0		101		3209		4064		1276		330		193		173		841		1960		3485	
	Recovered	0		0		1586		19,832		27,789		19,907		33,786		13,114		21,161		61,771		105,643		189,043	
**Mexico (N=127,792,286)**																									
	Confirmed	0		4		1211		18,009		71,440		135,425		198,548		174,923		143,656		181,746		181,746		312,551	
	Deaths	0		0		29		1830		8071		17,839		18,919		17,726		13,232		14,107		14,107		19,867	
	Recovered	0		0		35		11,388		52,349		110,766		152,577		169,107		131,785		151,364		151,364		251,209	
**Brazil (N=212,559,409)**																									
	Confirmed	0		2		5715		81,470		81,470		887,192		1,260,444		1,245,787		902,663		724,670		800,273		1,340,095	
	Deaths	0		0		201		5805		5805		30,280		32,881		28,906		22,571		15,932		13,236		21,829	
	Recovered	0		0		127		35,808		35,808		581,763		1,220,536		1,259,737		1,006,183		730,387		592,641		1,251,042	
**Argentina (N=45,195,777)**																									
	Confirmed	0		0		1054		3374		12,423		47,679		126,772		226,433		333,266		415,923		257,609		200,981	
	Deaths	0		0		27		191		321		768		2236		5117		8277		14,065		7728		4515	
	Recovered	0		0		240		1016		4080		16,692		61,752		217,415		293,450		379,294		283,288		169,449	
**Chile (N=19,116,209)**																									
	Confirmed	0		2		2842		14,858		105,848		155,843		76,274		56,059		51,265		47,265		41,487		57,230	
	Deaths	0		0		12		215		827		4634		3769		1832		1452		1466		1203		1198	
	Recovered	0		0		156		8424		34,147		198,502		87,098		55,552		52,710		50,053		39,962		50,778	
**United Kingdom (N=67,886,004)**																									
	Confirmed	2		59		38,754		139,956		78,768		27,677		19,577		33,290		117,763		558,947		618,940		862,498	
	Deaths	0		0		2457		24,297		10,773		2952		795		315		644		4412		11,900		15,077	
	Recovered	0		8		171		680		331		180		69		243		691		466		731		1909	
**France (N=65,273,512)**																									
	Confirmed	5		95		52,727		114,472		21,710		13,054		23,134		93,789		285,045		808,678		864,165		400,792	
	Deaths	0		2		3530		20,847		4426		1041		422		372		1346		4840		15,993		11,940	
	Recovered	0		12		9501		39,963		18,997		7926		5365		5026		11,842		24,463		44,818		32,229	
**Greece (N=10,423,056)**																									
	Confirmed	0		4		1310		1277		326		492		1068		5840		8158		20,776		66,020		33,579	
	Deaths	0		0		49		91		35		17		14		60		125		235		1780		2432	
	Recovered	0		0		52		1322		0		0		0		2430		7882		11,388		0		70,690	
**Taiwan (N=23,816,775)**																									
	Confirmed	9		29		283		107		13		5		20		21		26		41		120		124	
	Deaths	0		1		4		1		1		0		0		0		0		0		0		0	
	Recovered	0		9		30		283		101		14		3		22		21		32		50		106	
**Thailand (N=69,799,978)**																									
	Confirmed	17		23		1609		1303		127		90		139		107		152		215		224		3155	
	Deaths	0		0		10		44		3		1		0		0		1		0		1		3	
	Recovered	5		23		314		2342		279		93		69		149		105		213		219		462	
**South Korea (N=51,269,183)**																									
	Confirmed	10		3139		6636		988		729		1347		1486		5846		3707		2746		8017		27,117	
	Deaths	0		16		146		86		23		11		19		23		91		51		60		391	
	Recovered	0		27		5381		3664		1350		1191		1620		1965		6468		2691		3528		15,068	
**India (N=1,380,004,385)**																									
	Confirmed	1		2		1394		33,466		155,746		394,872		1,110,507		1,995,178		2,621,418		1,871,498		1,278,727		803,865	
	Deaths	0		0		35		1119		4254		11,992		19,111		28,777		33,390		23,433		15,510		11,117	
	Recovered	0		3		120		8945		82,784		256,060		746,462		1,745,508		2,433,319		2,218,312		1,398,072		970,695	
**Australia (N=25,459,700)**																									
	Confirmed	9		16		4534		2207		436		718		9360		8539		1277		499		317		513	
	Deaths	0		0		18		75		10		1		97		456		231		19		1		1	
	Recovered	2		9		347		5384		876		422		2943		11,367		3434		552		266		160	
**Egypt (N=102,334,403)**																									
	Confirmed	0		1		709		4827		4827		43,326		25,767		4861		4259		4357		8356		22,151	
	Deaths	0		0		46		346		346		1994		1852		616		509		336		384		981	
	Recovered	0		1		156		1224		1224		12,423		21,178		33,291		23,565		2958		3266		9387	

^a^Total population in 2020.

^b^The number of recovered cases after December 14, 2020, were not recorded in the COVID-19 Data Repository database (the record only includes cases from December 1 to 14, 2020); therefore, this value was underreported.

**Table 2:** Forecasting performance for each model in the validation set for the 15 countries.

Country (total population^a^) and methods		Mean error^b^	Root mean square error^b^	Mean absolute error^b^	Mean percentage error^b^	Mean absolute percentage error^b^
**United States (N=329,466,283)**						
	ARIMA^c^	–183,472.5153	229,501.345	183,888.691	–0.9538265	0.9562102
	FNN^d^	–197,967.69975	251,014.19	201,574.807	–1.027988	1.048648
	MLP^e^	34,016.71589	45,932.609	35,569.561	0.1774821	0.1862749
	LSTM^f^	–17,670.38	*41,667.98* ^g^	*31,092.06*	–0.09409045	*0.1664009*
**Canada (N=37,855,702)**						
	ARIMA	–3786.81463	4953.7659	3786.8146	–0.6828342	0.6828342
	FNN	–1902.8218773	3146.8161	2133.5721	–0.3503041	0.3898707
	MLP	–6056.7104430	7294.1933	6056.7104	–1.094643	1.094643
	LSTM	306.1702	*2272.551*	*1501.248*	0.04896196	*0.2723075*
**Mexico (N=127,792,286)**						
	ARIMA	–3776.6237	6281.987	4841.2544	0.3501243	1.2391347
	FNN	–15,894.200241	19,622.066	16,156.1290	–1.145524	1.165534
	MLP	–3551.381635	6534.119	5455.281	–0.2517612	0.3969063
	LSTM	–1137.118	*2883.836*	*2334.178*	–0.08386455	*0.1716616*
**Brazil (N=212,559,409)**						
	ARIMA	–52,913.8661	69,053.95	54,328.55	–0.7032164	0.7228866
	FNN	–168,251.54394	204,577.061	168,251.544	–2.240681	2.240681
	MLP	–28,723.33938	43,395.965	31,117.856	–0.3797225	0.412664
	LSTM	–2746.457	*16,085.02*	*14,347.73*	–0.03768765	*0.1931052*
**Argentina (N=45,195,777)**						
	ARIMA	10,240.495912	12,832.6035	10,240.4959	0.6433934	0.6433934
	FNN	22,285.962404	26,555.128	22,285.9624	1.402042	1.402042
	MLP	10,914.143275	13,689.5539	10,929.6874	0.6857769	0.6867919
	LSTM	1253.045	*3920.961*	*3202.607*	0.07803485	*0.2024643*
**Chile (N=19,116,209)**						
	ARIMA	1823.55216	1992.35	1823.5522	0.3048502	0.3048502
	FNN	8171.7723060	9157.9881	8171.7723	1.363951	1.363951
	MLP	2169.702307	2435.4540	2169.7023	0.3622628	0.3622628
	LSTM	595.9308	*790.8397*	*648.5224*	0.1001373	*0.1090634*
**United Kingdom (N=67,886,004)**						
	ARIMA	40,161.7481	55,436.735	41,580.2155	1.7053944	1.776331
	FNN	–17,129.950943	23,936.144	17,129.951	–0.7304511	0.7304511
	MLP	81,031.84	102,155.3238	81,031.841	3.482155	3.482155
	LSTM	15,560.98	*17,735.29*	*15,560.98*	0.6832804	*0.6832804*
**France (N=65,273,512)**						
	ARIMA	1807.5070	*8181.384*	*6633.665*	0.07287266	*0.2565254*
	FNN	61,075.99023	67,684.575	61,075.990	2.340844	2.340844
	MLP	9601.594851	11,456.382	10,239.308	0.3726648	0.3969022
	LSTM	6262.693	9254.264	7784.804	0.241549	0.3000627
**Greece (N=10,423,056)**						
	ARIMA	5423.2143	6072.0773	5423.2143	4.003338	4.003338
	FNN	–21.8694361	*561.98452*	*400.61927*	–0.01977488	*0.2937978*
	MLP	–1145.165405	1341.1596	1145.1654	–0.844399	0.844399
	LSTM	–512.1191	565.7909	512.1191	–0.3821559	0.3821559
**Taiwan (N=23,816,775)**						
	ARIMA	–15.97434477	17.288501	15.97434	–2.0379969	2.037997
	FNN	–6.571007146	7.379679	6.571007	–0.84606232	0.8460623
	MLP	–9.485179	12.925238	9.9162023	–1.2005706	1.257011
	LSTM	–2.059649	*3.322996*	*2.978151*	–0.3227033	*0.3820354*
**Thailand (N=69,799,978)**						
	ARIMA	1471.082153	1620.87009	1471.082153	23.7842238	23.784224
	FNN	1463.109910	1611.239573	1463.109910	23.659524	23.659524
	MLP	1517.21984066	1674.585004	1517.219841	24.5165025	24.516502
	LSTM	173.2286	*308.695*	*202.2714*	2.950519	*3.435209*
**South Korea (N=51,269,183)**						
	ARIMA	–260.265311	317.53169	265.29603	–0.4540395	0.4641688
	FNN	–75.7162332	*181.29894*	*154.2065*	–0.1226205	*0.2708482*
	MLP	–1138.0352476	1419.83911	1145.57606	–1.963196	1.978379
	LSTM	323.9709	342.9156	323.9709	0.5978793	0.5978793
**India (N=1,380,004,385)**						
	ARIMA	19,113.77834	21,947.375	19,113.778	0.1874688	0.1874688
	FNN	–10,156.962689	*13,612.018*	*10,156.963*	–0.09945817	*0.09948717*
	MLP	20,964.3576266	24,556.936	20,964.358	0.2055718	0.20055718
	LSTM	–13,037.64	14,480.91	13,037.64	–0.128178	0.1281378
**Australia (N=25,459,700)**						
	ARIMA	26.9606020	30.40208	26.96060	0.09542063	0.09542063
	FNN	187.8959192	205.6998	187.89592	0.6634038	0.6637038
	MLP	–15.69085695	76.48186	62.261210	–0.05478576	0.2197826
	LSTM	5.898776	*14.39023*	*11.91991*	0.02086999	*0.04212132*
**Egypt (N=102,334,403)**						
	ARIMA	2392.285714	3239.04732	2392.28571	1.7844594	1.784459
	FNN	1944.5586880	2641.98168	1944.55869	1.45017	1.45017
	MLP	669.96030638	936.05245	669.96031	0.4988667	0.4988667
	LSTM	437.0412	*500.6487*	*438.0092*	0.3304228	*0.3311979*

^a^Total population in 2020.

^b^Five commonly used measures for evaluation of forecasting include mean error, root mean square error (RMSE), mean absolute error (MAE), mean percentage error, and mean absolute percentage error (MAPE), according to the records of the latest 14 days in 2020. The RMSE, MAE, and MAPE are always positive values.

^c^ARIMA: autoregressive integrated moving average.

^d^FNN: feedforward neural network.

^e^MLP: multilayer perceptron.

^f^LSTM: long short-term memory.

^g^The values for best performances in each country are italicized.

## Discussion

### Principal Findings

A combination of data on COVID-19 incidence and policy measures can be used to examine the relationship between the progression of the COVID-19 pandemic and governmental epidemic prevention efforts. The CPAIS can help users determine whether policy measures are successful in preventing COVID-19 transmission. According to a report published by the Lowy Institute for International Policy [53], a ranked comparison of the performance of countries in managing the COVID-19 pandemic shows that New Zealand, Vietnam, and Taiwan are the top three countries with the highest average scores on their six indicators. Besides, New Zealand and Taiwan successfully controlled the COVID-19 outbreak without international financial support (Figures S1-S3 in Multimedia Appendix 1). Specifically, New Zealand had immediately implemented infection control and closure policies with a flexible adaptation on measures; in addition, Taiwan had enforced strict guidelines regarding international travel that not only contributed to infection control but also rendered the strict measures described in the containment and closure policies unnecessary. Furthermore, both countries had taken great efforts to maximize the implementation of testing and contact tracing policies during 2020. In this regard, both countries are outstanding examples. The vivid heat maps in the CPAIS illustrate time-dependent fluctuations in the measures and help users monitor variations in, and the effects of, policy measures in each country.

Several time series AI learning techniques have been used for forecasting purposes. Both statistical learning and deep learning models demonstrated efficacious performance for different countries. Although the values are not absolute, they are comparable between countries with different total populations. When compared to the results of a past study [19], performance for the same model and country was better in this study because more extensive time series data were included in our system. In addition, 14-day COVID-19 trend forecasting can serve a useful alert that will help governments and experts reduce the incidence of COVID-19. Furthermore, different AI learning techniques have unique advantages.

According to the Wold decomposition theorem [34,54,55], the autoregressive moving average model is theoretically sufficient to describe a regular stationary time series. It is possible to change a nonstationary time series into a stationary one, such as by using differencing. As noted earlier, ARIMA models have three components: autoregression, integration, and moving average. They are applied to data with evidence of nonstationarity in the mean, whereby an initial differencing step can be applied one or more times to eliminate the nonstationarity of the mean function in the trend. We used the *auto.arima* function for R to choose the best model according to either the Akaike information criterion, corrected Akaike information criterion, or Bayesian information criterion value; the *auto.arima* function also conducts the model search within the order constraints provided. FNN is similar to ARIMA because the fitted model is analogous to an autoregression(p) model, where p is the order but with nonlinear functions for nonseasonal data in this study. Therefore, it is denoted as a neural network autoregression(p,k) model called *NNAR*, where k represents the number of hidden nodes. That is why, for some countries, ARIMA and FNN yielded similar outcomes for forecast accuracy. Differences between the two models still exist; the error can be reduced only for FNN by increasing the number of iterations, but the iteration time will be increased as a result.

The capabilities of neural networks are attributable to the hierarchical or multilayered structure of the networks. The data structure can include features at different scales or resolutions and combine them into higher-order features. After repeating the learning process for a sufficient number of training cycles, the network will transition to some state where the error term is small enough. Generalization and tolerance are the two main characteristics. First, neural networks permit generalization because they can classify both unknown and known patterns with the same distinguishing features. Second, neural networks are highly fault tolerant. Because of their distributed nature, they will continue to function even if a significant fraction of neurons and interconnections fail. In general, increasing the number of hidden nodes may enhance the performance of prediction, and increasing the number of networks to train may result in an ensemble forecast.

**Figure 6:**
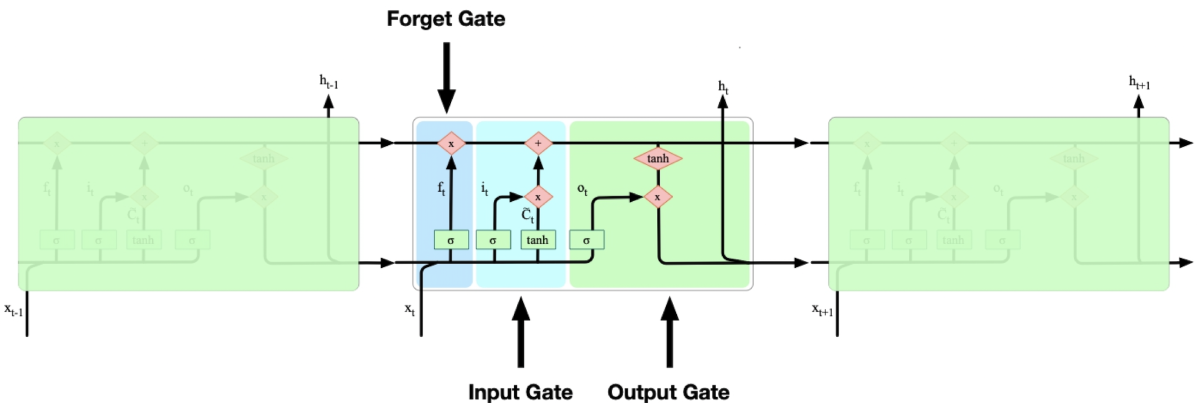
Diagram of the long short-term memory neural network with three functional gates.

The core idea of LSTM lies in the cell state—the horizontal line that runs down the chain with information flowing alongside (Figure 6). In addition, LSTMs have the ability to remove or add information to the cell state, controlled by the gates, which are a pathway through which information can be allowed to pass. They consist of a sigmoid neural net layer and a pointwise multiplication operation. LSTM networks are powerful in promptly forecasting series data since there can be lags of unknown duration between events in time series. Hence, when compared to other traditional RNNs in this study, LSTM networks do not have the vanishing gradient problem. Thus, LSTM has the advantages of being relatively insensitive to time intervals and of making fewer errors in prediction when compared to other methods.

In the CPAIS, long-term cumulative records of confirmed cases, recoveries, and deaths are included. In addition, daily figures for these metrics are provided for each month. Thus, short-term trends can be examined using this system. Users can compare three or more countries and visualize the relative incidence of COVID-19 within a specific time duration. Short-term and long-term trends can be simultaneously viewed. In previous studies [14,19,20], only a limited number of countries were included for forecasting. Our system contains 171 countries and provides information about policy measures. Further, data visualization, statistical and deep learning for incidence forecasting, and customized ranking are possible. Based on their objectives, users can select country names and time periods. Similar cultural backgrounds, neighboring geographical characteristics, and high-frequency trading may also serve as attractive features. In particular, a declined ranking is calculated by our system to explore the effectiveness of COVID-19 management strategies implemented in 2020. Thus, the CPAIS is a comprehensive AI-based service that is available on the internet. It relies on big data and offers data visualization, deep learning–based prediction, and customized comparison. This system can be used to investigate COVID-19 progression trends.

### Limitations

To the best of our knowledge, this is the first web-based machine learning system that can explore variations, trends, and forecasts related to the COVID-19 pandemic across 171 countries. This pilot system still has several limitations. First, this database relies heavily on the source databases and shares similar limitations with the source databases. For example, the source databases did not consider the number of COVID-19 patients that were traveling internationally, and this may result in inaccurate analysis for a small number of countries. However, we think that the number of COVID-19 patients who were traveling internationally is small, as most countries imposed COVID-19–negative tests or proof of vaccination before allowing the traveler into the country. Second, the CPAIS cannot be updated daily if the source databases are not updated. For example, at present, the number of recoveries in the United States was last updated on December 14, 2020. So the number of recoveries in the United States may not be accurate. Finally, since the main purpose of this platform is to consolidate raw data retrieved from various databases and associated measures of pandemic policy implementation, we remind the reader to use text mining, local reports, and information retrieved from the medical system of a given country for further assessment.

### Conclusions

In general, the CPAIS collects and summarizes information about the COVID-19 pandemic and offers data visualization and deep learning–based prediction. It may be a useful and consequential reference resource for any serious outbreak or epidemic that may occur in the future. In addition, information about the vaccine is also stored in our system. It may be used to evaluate the efficacy of the vaccine in different countries in the future. Moreover, the 2-week machine learning forecasts may serve as warning signs and highlight current trends in the epidemic that have been made apparent by AI techniques. To conclude, the CPAIS can be used to summarize several factors that can influence the effectiveness of epidemic prevention and predict the next serious outbreak.

## Appendix

Multimedia Appendix 1 Hyperparameters, packages, and function code for each model as well as the dynamic heat maps with policy measures for New Zealand, Vietnam, and Taiwan.

## Abbreviations

AI: artificial intelligence
ARIMA: autoregressive integrated moving average
CPAIS: COVID-19 Pandemic AI System
CSSE: Center for Systems Science and Engineering
FNN: feedforward neural network
LSTM: long short-term memory
MAE: mean absolute error
MAPE: mean absolute percentage error
ME: mean error
MLP: multilayer perceptron
MPE: mean percentage error
NNAR: neural network autoregression model
OxCGRT: Oxford COVID-19 Government Response Tracker
RMSE: root mean square error
RNN: recurrent neural network
Sass: Syntactically Awesome Style Sheets
SQL: Structured Query Language
WHO: World Health Organization
