# Correction: A COVID-19 Pandemic Artificial Intelligence–Based System With Deep Learning Forecasting and Automatic Statistical Data Acquisition: Development and Implementation Study

**DOI:** 10.2196/31085

**Published:** 2021-07-09

**Authors:** Cheng-Sheng Yu, Shy-Shin Chang, Tzu-Hao Chang, Jenny L Wu, Yu-Jiun Lin, Hsiung-Fei Chien, Ray-Jade Chen

**Affiliations:** 1 Department of Family Medicine School of Medicine, College of Medicine Taipei Medical University Taipei Taiwan; 2 Department of Family Medicine Taipei Medical University Hospital Taipei Taiwan; 3 Graduate Institute of Biomedical Informatics College of Medical Science and Technology Taipei Medical University Taipei Taiwan; 4 Professional Master Program in Artificial Intelligence in Medicine College of Medicine Taipei Medical University Taipei Taiwan; 5 Clinical Big Data Research Center Taipei Medical University Hospital Taipei Taiwan; 6 Division of Plastic Surgery, Department of Surgery Taipei Medical University Hospital and School of Medicine, College of Medicine Taipei Medical University Taipei Taiwan; 7 Department of Surgery School of Medicine, College of Medicine Taipei Medical University Taipei Taiwan; 8 Division of General Surgery Department of Surgery Taipei Medical University Hospital Taipei Taiwan

In “A COVID-19 Pandemic Artificial Intelligence–Based System With Deep Learning Forecasting and Automatic Statistical Data Acquisition: Development and Implementation Study” (J Med Internet Res 2021;23(5):e27806), the authors noticed two errors.

In the originally published manuscript, authors Cheng-Sheng Yu and Shy-Shin Chang should have been denoted as having contributed equally to the paper but were not. A footnote clarifying equal contribution has now been added to each of the aforementioned authors.

Additionally, in the originally published manuscript, the Acknowledgments section was omitted. In the corrected version, a new Acknowledgments section has been added with the following statement:

This study is supported by the Ministry of Science and Technology Grant (MOST 110-2314-B-038-025) and Higher Education Sprout Project by the Ministry of Education (MOE) in Taiwan (DP2-110-21121-01-A-09). No funding bodies had any role in study design, data collection and analysis, decision to publish, or preparation of the article.

The correction will appear in the online version of the paper on the JMIR Publications website on July 9, 2021, together with the publication of this correction notice. Because this was made after submission to PubMed, PubMed Central, and other full-text repositories, the corrected article has also been resubmitted to those repositories.

