# Supplementary material for: A COVID-19 Pandemic Artificial Intelligence–Based System With Deep Learning Forecasting and Automatic Statistical Data Acquisition: Development and Implementation Study
Source: J Med Internet Res. 2021 May 20;23(5):e27806. doi: 10.2196/27806 (PMC8139395; doi:10.2196/27806)

Multimedia Appendix 1

Table S1. The information of hyperparameters, packages and function code for each model

Models	Hyperparameter	Library Package and Code
Autoregressive Integrated Moving Average	Returns best ARIMA model according to either AIC, AICc or BIC value.<br><br>e.g. Greece data<br>order = c(5, 2, 2)<br>seasonal = c(0, 0, 2)<br><br>lambda="auto"<br><br>frequency = 14	library(forecast)<br>auto.arima(y,…)<br>Arima(y,<br>order = c(0, 0, 0),<br>seasonal = c(0, 0, 0),<br>xreg = NULL,<br>include.mean = TRUE,<br>include.drift = FALSE,<br>include.constant,<br>lambda = model$lambda,<br>biasadj = FALSE,<br>method = c("CSS-ML", "ML", "CSS"),<br>model = NULL,<br>x = y, ...)
Feedforward Neural Network	NNAR(1,1,50)<br>p =1, P = 1, size=50<br><br>maxit=100<br><br>frequency = 14	library(forecast)<br>nnetar(y,<br>p,<br>P = 1,<br>size,<br>repeats = 20,<br>xreg = NULL,<br>lambda = NULL,<br>model = NULL,<br>subset = NULL,<br>scale.inputs = TRUE,<br>x = y, ...)
Multilayer Perceptron Neural Networks (MLPs)	m = frequency = 14<br><br>rep=20<br><br>hd=c(5,5,5)	library(nnfor)<br>mlp(y, m = frequency(y), hd = NULL, reps = 20, comb = c("median", "mean", "mode"), lags = NULL, keep = NULL, difforder = NULL, outplot = c(FALSE, TRUE), sel.lag = c(TRUE, FALSE), allow.det.season = c(TRUE, FALSE), det.type = c("auto", "bin", "trg"), xreg = NULL, xreg.lags = NULL, xreg.keep = NULL,

		hd.auto.type = c("set", "valid", "cv", "elm"), hd.max = NULL, model = NULL, retrain = c(FALSE, TRUE), ...)
Long Short-term Memory (LSTM)	X_shape2 = 1<br>X_shape3 = 1<br>batch_size = 1<br>units = 1<br>scaler = c(0, 3316)	library(caret)<br>library(keras)<br>library(tensorflow)<br>dim(x_train) <- c(length(x_train), 1, 1)<br>batch_size = 1<br>units = 1<br><br>model <- keras_model_sequential()<br>model%>%<br>layer_lstm(units, batch_input_shape = c(batch_size, X_shape2, X_shape3), stateful= TRUE)%>%<br>layer_dense(units = 1)<br><br>#Compile the model<br>model %>% compile(<br>loss = 'mean_squared_error',<br>optimizer = optimizer_adam(lr= 0.02, decay = 1e-6), metrics = c('accuracy'))<br><br>Epochs = 50<br>for(i in 1:Epochs){<br>model %>% fit(x_train, y_train, epochs=1, batch_size=batch_size, verbose=1, shuffle=FALSE)<br>model %>% reset_states()<br>}

Figure S1. The dynamic heatmap with policy measures for New Zealand

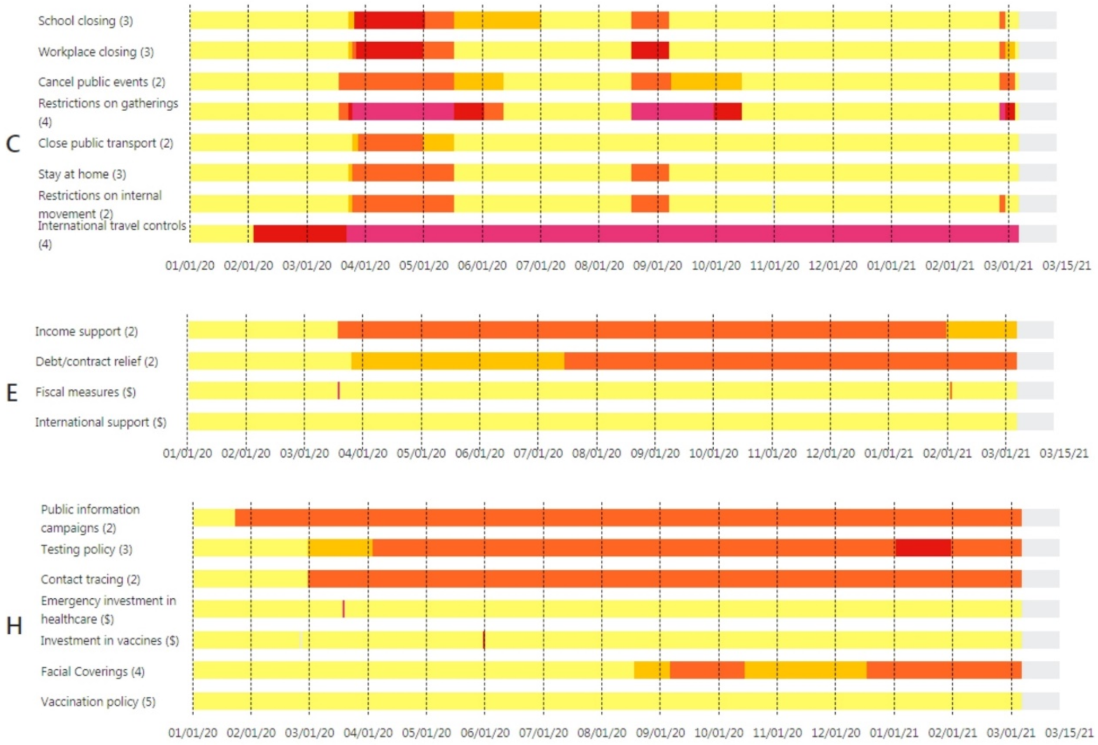

Figure S2. The dynamic heatmap with policy measures for Vietnam

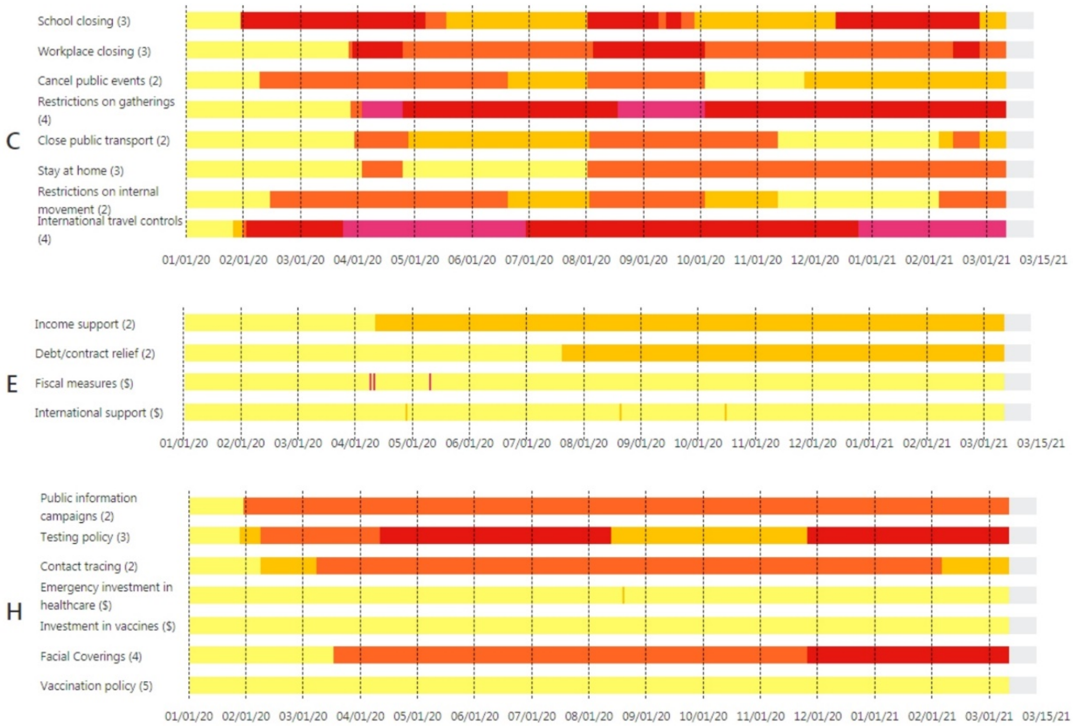

Figure S3. The dynamic heatmap with policy measures for Taiwan

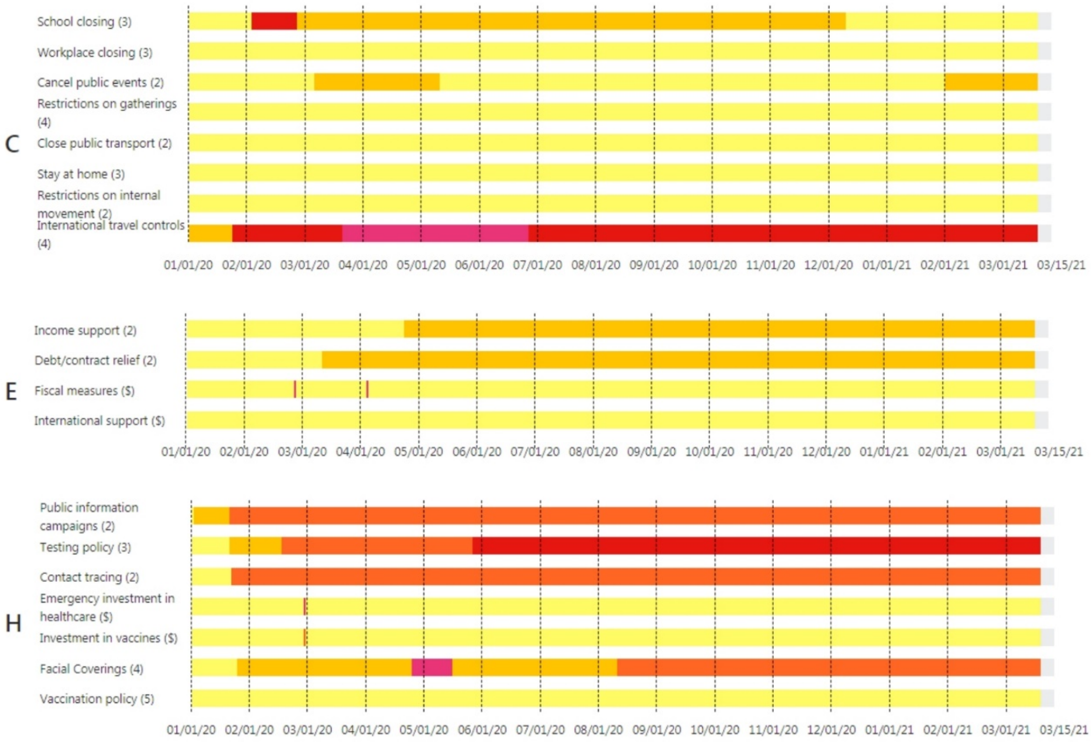

Supplement: Multimedia Appendix 1 [file jmir_v23i5e27806_app1.pdf]
